# Graphene oxide containing self-assembling peptide hybrid hydrogels as a potential 3D injectable cell delivery platform for intervertebral disc repair applications

**DOI:** 10.1016/j.actbio.2019.05.004

**Published:** 2019-07-01

**Authors:** Cosimo Ligorio, Mi Zhou, Jacek K. Wychowaniec, Xinyi Zhu, Cian Bartlam, Aline F. Miller, Aravind Vijayaraghavan, Judith A. Hoyland, Alberto Saiani

**Affiliations:** a School of Materials, Faculty of Science and Engineering, The University of Manchester, Oxford Road, Manchester M13 9PL, UK; b Manchester Institute of Biotechnology (MIB), The University of Manchester, Oxford Road, Manchester M13 9PL, UK; c School of Chemical Engineering and Analytical Sciences, Faculty of Science and Engineering, The University of Manchester, Oxford Road, Manchester M13 9PL, UK; d National Graphene Institute (NGI), The University of Manchester, Oxford Road, Manchester M13 9PL, UK; e Division of Cell Matrix Biology and Regenerative Medicine, School of Biological Sciences, Faculty of Biology, Medicine and Health, The University of Manchester, Oxford Road, Manchester M13 9PL, UK; f NIHR Manchester Biomedical Research Centre, Manchester University NHS Foundation Trust, Manchester Academic Health Science Centre, Grafton St, M13 9WU Manchester, UK

**Keywords:** Peptide, Graphene oxide, Hydrogels, Injectable, Nucleus pulposus, Cell delivery, Cell culture, Tissue engineering

## Abstract

Cell-based therapies have shown significant promise in tissue engineering with one key challenge being the delivery and retention of cells. As a result, significant efforts have been made in the past decade to design injectable biomaterials to host and deliver cells at injury sites. Intervertebral disc (IVD) degeneration, a major cause of back pain, is a particularly relevant example where a minimally-invasive cellular therapy could bring significant benefits specifically at the early stages of the disease, when a cell-driven process starts in the gelatinous core of the IVD, the nucleus pulposus (NP).

In this present study we explore the use of graphene oxide (GO) as nano-filler for the reinforcement of FEFKFEFK (β-sheet forming self-assembling peptide) hydrogels. Our results confirm the presence of strong interactions between FEFKFEFK and GO flakes with the peptide coating and forming short thin fibrils on the surface of the flakes. These strong interactions were found to affect the bulk properties of hybrid hydrogels. At pH 4 electrostatic interactions between the peptide fibres and the peptide-coated GO flakes are thought to govern the final bulk properties of the hydrogels while at pH 7, after conditioning with cell culture media, electrostatic interactions are removed leaving the hydrophobic interactions to govern hydrogel final properties. The GO-F820 hybrid hydrogel, with mechanical properties similar to the NP, was shown to promote high cell viability and retained cell metabolic activity in 3D over the 7 days of culture and therefore shown to harbour significant potential as an injectable hydrogel scaffold for the in-vivo delivery of NP cells.

**Statement of Significance:**

Short self-assembling peptide hydrogels (SAPHs) have attracted significant interest in recent years as they can mimic the natural extra-cellular matrix, holding significant promise for the *ab initio* design of cells’ microenvironments. Recently the design of hybrid hydrogels for biomedical applications has been explored through the incorporation of specific nanofillers. In this study we exploited graphene oxide (GO) as nanofiller to design hybrid injectable 3Dscaffolds for the delivery of nucleus pulposus cells (NPCs) for intervertebral disc regeneration. Our work clearly shows the presence of strong interactions between peptide and GO, mimicking the mechanical properties of the NP tissue and promoting high cell viability and metabolic activity. These hybrid hydrogels therefore harbour significant potential as injectable scaffolds for the in vivo delivery of NPCs.

## Graphical abstract

**Figure:**
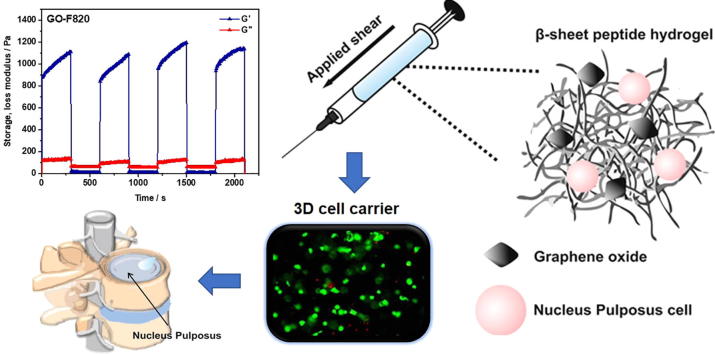

## 1 Introduction

Cell-based therapies have shown significant promise in tissue engineering [1]. The aim of this approach is to deliver specific cell(s) to injury site(s) in order to promote regeneration of the concerned tissue(s). Significant advances in cell engineering have resulted in the availability of a large number of cell types for the repair of a large variety of tissues but the delivery of these cells remains a challenge. It has been shown that delivery using cells suspended in solution results in poor cell retention at injury site as well as poor cell survival [2]. As a result, significant efforts have been made to design suitable biomaterials that allow the delivery and retention of cells at injury site through minimal invasive approaches such a direct injection.

Intervertebral disc (IVD) degeneration, a major cause of low back pain [3], [4], is a particularly relevant example where a minimally-invasive cellular therapy could bring significant benefits in particular at the early stages of the disease. IVD is composed of an inner gelatinous core, the nucleus pulposus (NP), surrounded by a tough fibrous tissue the annulus fibrosis (AF) [5]. IVD degeneration is a cell-driven process that starts in the NP [6]. It results from a change in the physiochemical cellular environment that leads NP cells to produce catabolic and degradative enzymes causing progressive tissue degeneration, loss of disc height and ultimately pain [7], [8], [9], [10], [11], [12]. Due to the avascular nature of the NP and the low mitotic activity of NP cells, the IVD has a low self-healing ability. Several groups are currently developing minimally-invasive cell-based therapies to restore NP cell population and regenerate the NP [13], [14], [15], [16]. For example recently transplantation of NP cells was shown to retard IVD degeneration in a dog model, and to significantly reduce lower back pain, with retention of disc height and hydration level, in a randomised human clinical trial [17].

As far as cell delivery vehicles for the NP are concerned, injectable hydrogels have attracted significant interest. Indeed, in addition to the delivery and retention of cells, hydrogels allow the mechanical properties and highly hydrated nature of the NP to be recapitulated with the aim to preserve and/or restore its mechanical functionality. One particular family of hydrogels that has attracted significant attention in the last decade due to their biocompatible and non-immunogenic nature are peptide-based hydrogels [18], [19], [20]. A large variety of peptide designs have been developed for the formulation of peptide hydrogels for numerous biomedical applications including injectable self-healing systems for cell delivery [21]. One of the most popular designs was devised by Zhang and co-workers and is based on the self-assembling of short β-sheet forming peptides (typically 8–20 amino acids long with alternating hydrophilic and hydrophobic residues) into extended cross-β-sheet fibres that entangle/associate to form 3 dimensional fibrillar networks swollen by water i.e: hydrogels [22], [23]. This family of hydrogels have been shown to be suitable for the culture of a several cell types [24], [25], [26], [27], including NP cells [28]. One recent approach developed to tailor the properties and functionality of peptide based hydrogels has been the use of nanofillers, in particular carbon based nanofillers [29]. Graphene oxide (GO) stands out due to its high water dispersibility, good biocompatibility and promotion of cell adhesion [30], [31], [32]. In addition, different strategies have been developed to functionalise GO making it a potential vehicle for the functionalisation of peptide hydrogels [33], [34], [35].

In this work we explore the use of GO as a nanofiller for the design of hybrid peptide hydrogels for the delivery of NP cells. For this purpose, we first investigated the effect of adding GO has on the physico-chemical properties of the hydrogels. Subsequently NP cells were encapsulated and cultured over 7 days to demonstrate the hybrid hydrogels potential suitability as scaffold for their delivery.

## 2 Materials and methods

### 2.1 Materials

FEFKFEFK peptide (HCl salt) was purchased from Biomatik Corporation (Wilmington, DE, Canada). The peptide purity (94%) was confirmed in-house by mass spectroscopy (MS) and reverse phase high performance liquid chromatography (HPLC). All solvents and reagents were purchased from Sigma-Aldrich and used as received.

### 2.2 Graphene oxide (GO) preparation and characterisation

GO used in this study was prepared by a modified Hummers method [36]. Briefly, graphite (10 g, 80 mesh, 94% carbon) was first treated with NaNO_3_ (9 g) and concentrated H_2_SO_4_ (338 mL) at room temperature (RT) for 3 h to obtain intercalated graphite. The mixture was then cooled in an ice bath and 45 g of KMnO_4_ gradually added. After addition of the oxidizing agent, the reaction mixture was stirred at room temperature for one week to complete the oxidation. The oxidised graphitic slurry was then diluted with a solution of 5% H_2_SO_4_, followed by slow addition of 5 g of H_2_O_2_ as solution. The resulting GO was purified by repeated centrifugation and re-dispersion in deionised (DI) water until the pH of the supernatant was neutral. The size distribution of GO flakes was characterised using scanning electron microscopy (Zeiss Ultra FEG SEM). GO flakes (50 µg/mL^−1^) were spin-coated onto a plasma-treated Si/SiO_2_ wafer at 3500 rpm and acceleration of 350 rpm/s for 5 mins to get monolayer coverage with minimal flake overlap and left to dry overnight before imaging. For each dispersion the size of ∼300 flakes from three SEM images was measured manually using ImageJ® software. In order to avoid shape anisotropies to skew the distribution results, measurements were performed in the horizontal direction through the centre of the flake assuming that the drying process did not introduce any orientation anisotropy.

### 2.3 Peptide and peptide/GO hydrogel formulation

Depending on final concentration targeted, the desired amount of peptide powder was dissolved in HPLC grade water. The hydrogels were then prepared by adjusting the pH to 4 by of a 0.5 M NaOH solution. When hybrid hydrogels were prepared GO was added as a 3 mg mL^−1^ aqueous solution after NaOH addition to prevent GO precipitation, as observed previously [37]. Finally the total sample volume was adjusted by further addition of HPLC grade water to achieve the desired concentration. All hydrogels were sterilised with UV C pulsed light (3 pulses of 300 mJ cm^−3^ per sample) using a SteriBeam sterilization system. The name and formulation of the samples used in this study are listed in Table 1.

**Table 1:** Summary of sample names and formulations.

Sample name	Peptide concentration (mg mL^−1^)	GO concentration (mg mL^−1^)
F810	10	0
F815	15	0
F820	20	0
GO-F810	10	0.5
GO-F815	15	0.5
GO-F820	20	0.5

### 2.4 Attenuated total reflectance Fourier-transform infrared spectroscopy (ATR-FTIR)

Measurements were performed on a Thermo-Fisher Nicolet 5700 FTIR spectrophotometer equipped with an attenuated total reflectance (ATR) diamond accessory. 20 µL of hydrogel was pipetted onto the crystal surface of the spectrophotometer and the beam path purged with dry CO_2_-scrubbed air. Absorbance spectra were obtained using the average of 128 scan with a 4 cm^−1^ resolution. HPLC grade water background was subtracted from each spectra using the built-in OMNIC software provided with the instrument. All measurements were done in triplicate. The FTIR spectra were smoothed using a Savitzsky-Golay filter with a 9-points window. A baseline was subtracted in the Amide I region (1500–1700 cm^−1^) from the original spectra using OriginPro v8.5.1 software.

### 2.5 Atomic force microscopy (AFM)

Hydrogels were first diluted 10-fold using ddH_2_O. 50 µL of diluted hydrogel were then drop-cast on freshly-cleaved mica and left for 2 min. Excess solution was then removed from the surface of the specimens and the mica stubs rinsed 10-times by pipetting 100 µL of ddH_2_O directly onto the surface. Excess liquid was finally removed using Whatman No. 1 filter paper. Samples were left to air-dry overnight before imaging. AFM imaging was performed on a Bruker Multimode 8 instrument (Bruker, USA). Samples were scanned in air at room temperature in ScanAsyst mode, using a ScanAsyst-Air probe with a resonant frequency, *f*_0_, of 70 kHz and a nominal spring constant, *k*, of 0.4 N m^−1^. AFM images were acquired at a 512 × 512 pixels resolution over scanning areas ranging from 2 × 2 to 10 × 10 μm at a scan frequency of 1 Hz and analysed with Nanoscope Analysis 1.40 software.

### 2.6 Transmission electron microscopy (TEM)

Hydrogel samples were first diluted 10-fold using ddH_2_0. A carbon-coated copper grid (400 mesh, Electron Microscopy Sciences, UK) was then placed sequentially on a: 10 µL sample droplet for 1 min, 10 µL ddH_2_O droplet for 10 s, 10 μL 1% uranyl acetate (negative staining) solution droplet for 30 s, 10 µL droplet of ddH_2_O for 10 s. Excess liquid was removed following each step using a lint-free tissue. Samples were then left to air-dry before imaging. TEM images were obtained using a FEI Tecnai12 BioTwin at 100 KeV with Gatan Orius SC1000A CCD camera.

### 2.7 Oscillatory shear rheometry

Oscillatory shear rheometry was performed on a Discovery Hybrid 2 (DHR-2) rheometer (TA Instruments, USA) using a 20 mm parallel plate geometry with a gap size of 500 µm. Samples were prepared by pipetting 300 μL of hydrogel into ThinCert well inserts (1 μm pore size Greiner Bio-One Ltd, Gloucestershire, UK). The inserts were then placed into 12-well plates and incubated at 37 °C overnight in either 1 mL of ddH_2_O or bovine nucleus pulposus cell culture media. After media exposure, samples were removed from the inserts by peeling-off the bottom membrane of the insert and transferred onto the rheometer plate. The upper rheometer head was then lowered to the desired gap size and samples were left to equilibrate for 3 min at 37 °C. Frequency sweep experiments were performed at 0.2% strain, within the linear viscoelastic region in the frequency range: 0.01–10 Hz. For shear-thinning recovery experiments, samples were tested in time-sweep mode by alternating cycles of recovery (5 min at 0.2% strain and 1 Hz frequency) with cycles of high-shear (5 min at 100% strain and 1 Hz frequency). For all experiments a solvent trap was used to minimise sample evaporation. All measurements were repeated at least three times to ensure reproducibility.

### 2.8 Bovine nucleus pulposus cell culture and encapsulation

NP cells were isolated from bovine tails obtained from a local abattoir and expanded in monolayer culture using DMEM culture media (100 units ml^−1^ penicillin, 100 μg ml^−1^ streptomycin, 0.25 μg ml^−1^ amphotericin, 10 μM ascorbic acid 2-phosphate, 100 mM of sodium pyruvate) containing 10% (v/v) foetal calf serum. The cells were passaged at 70–80% confluency using a 1× trypsin/EDTA solution and the cell suspension collected in a 50 mL falcon tube. A cell pellet was then recovered from the cell suspension by centrifugation (350 g for 5 min) and re-suspended in fresh culture media to the desired cell density. Hydrogels were pre-warmed at 37 °C and bovine NP cells encapsulated by gentle pipetting and mixing 100 μL of cell suspension into 1 mL of hydrogel to a final cell density of 4 × 10^6^ cells mL^−1^ of hydrogel. After encapsulation, 100 μL aliquots of cell-laden hydrogels were dispensed in ThinCert well inserts and conditioned by adding 1 mL of cell culture media in each culture well. The cell culture plates were incubated at 37 °C and 5% CO_2_. Media was changed every 20 min during the first hour and every two days thereafter.

### 2.9 Assessment of cell viability

To assess cell viability a LIVE/DEAD membrane integrity assay as a surrogate measure of viability was used (Invitrogen, Thermo Fisher Scientific, UK). Media was removed from the cell culture wells and the hydrogels incubated for 1 h with a membrane integrity assay as a surrogate measure of viability LIVE/DEAD assay solution (10 mL phosphate buffer saline (PBS), 20 μL ethidium homodimer-1 and 5 μL of calcein AM). After incubation the LIVE/DEAD assay solution was gently removed and the cell-laden hydrogels transferred onto microscope glass slides for imaging. Cell images were collected using a Nikon Eclipse 50i fluorescence microscope (emission wavelengths: green channel live cells 515 nm; red channel dead cells 635 nm; excitation wavelength: 495 nm) at days 1, 4 and 7. Three samples per time point were used. The fluorescence composite images in red, green and blue (RGB) were split in their individual channels using ImageJ® (v.1.51) to produce three 8-bit greyscale images. Greyscale images were thresholded using Huang’s approach [38] and touching cells were separated into individual objects by applying a watershed algorithm [39]. Numbers of live and dead cells were obtained from the greyscale images of the green and red channels respectively using the ‘analyse particles’ plugin. Percentage viability was calculated as: 100 × [Number of alive cells/Total cell number (alive + dead)].

### 2.10 Assessment of metabolic activity

To assess cell metabolic activity, media was removed from the cell culture wells and the hydrogels incubated for 2.5  h with a 10% AlamarBlue DMEM solution at 37 °C and 5% CO_2_. After incubation the AlamarBlue solution was gently removed and transferred to a 96-well plate (100 μL per well) and fluorescence measured using a BioTek™ FLx800™ microplate fluorescence reader (excitation wavelength: 540 nm; emission wavelength: 590 nm). The fluorescence intensity measured was normalised using a blank hydrogels (without cells) fluorescence.

### 2.11 Cell clustering

To assess cell clustering the area distribution of the green fluorescent images obtained during LIVE/DEAD staining were used. The images were processed as described above and the greyscale images corresponding to the green channel used to create frequency plots of cell and cell clusters area. Cell areas were then obtained using the ‘analysing particles’ plugin on the greyscale image relative to the green channel and results were plotted as area-size-frequency plots. The cell area value below which 50% of the cell area distribution is contained (D50) was also obtained.

### 2.12 Statistical analysis

All quantitative values were presented as mean ± standard deviation. All experiments were performed using at least three replicates. Data were compared using one-way analysis of variance (ANOVA) implemented in Microsoft Excel 2016. Two level of significance: 0.05 (**) and 0.001 (***) were used.

## 3 Results and discussion

**Fig. 1:**
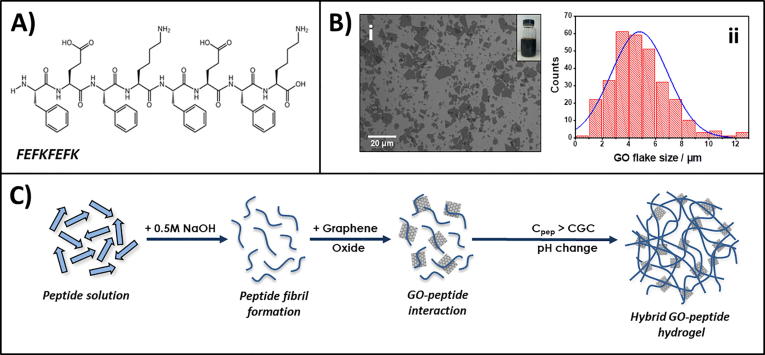
Top: Chemical structure of FEFKFEFK peptide (A), SEM image of GO flakes (Bi) and flake size distribution (Bii). Bottom: Schematic representation of the formulation route used to prepare peptide/GO hybrid hydrogels (C).

We recently have investigated the interactions between a range of graphene derivatives, including GO, and this family of β-sheet forming peptides [29]. For the purpose of this current work we selected the peptide FEFKFEFK (F: phenylalanine, E: glutamic acid, K: lysine – Fig. 1A). Two main reasons guided our choice: 1. This peptide will carry an overall positive net-charge at low pH (i.e.: during formulation of the hydrogels at pH 4 – Fig. ESI 1) while the surface of GO flakes will carry a negative net-charge [40]. We therefore expect electrostatic interactions between the peptides and the GO flakes to promote a good interfacial adhesion between fibres and flakes. 2. This type of peptide will carry no overall net-charge at neutral pH (i.e.: under cell culture conditions – Fig. ESI 1) which has been shown to allow the formulation of stable hydrogels suitable for the culture of cells with low mitotic activity such as chondrocytes [26] and NP cells [28]. The GO used in our study was synthesised using a modified Hummers method [36]. The flake mean-size was 4.8 ± 2.2 µm (Fig. 1B), smaller than the GO flakes used in our previous study, with surface oxidation level of 25% (Fig. ESI 2A–B). Hybrid hydrogels were formulated by adding GO in solution during hydrogel preparation as schematically shown in Fig. 1C.

**Fig. 2:**
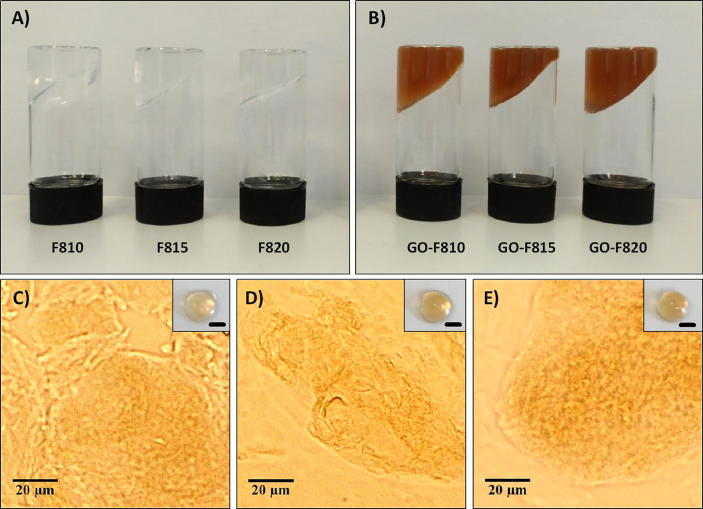
Top: Inverted vials photographs of peptide hydrogels (A) and peptide-GO hybrid hydrogels (B) taken one hour after formulation. Bottom: Light microscopy images of GO-F810 (C), GO-F815 (D) and GO-F820 (E) hydrogels (Inset: photographs of corresponding hydrogel droplets, 30 µL each – Scale bar 2 mm).

In Fig. 2A-B photographs of the hydrogels obtained with and without GO are presented. As can be seen optically homogeneous hydrogels were obtained in both cases with the hydrogel containing GO acquiring a typical brown colour. The quality of the GO dispersion was confirmed by optical microscopy, as no micron-size aggregates could be observed (Fig. 2C-E).

**Fig. 3:**
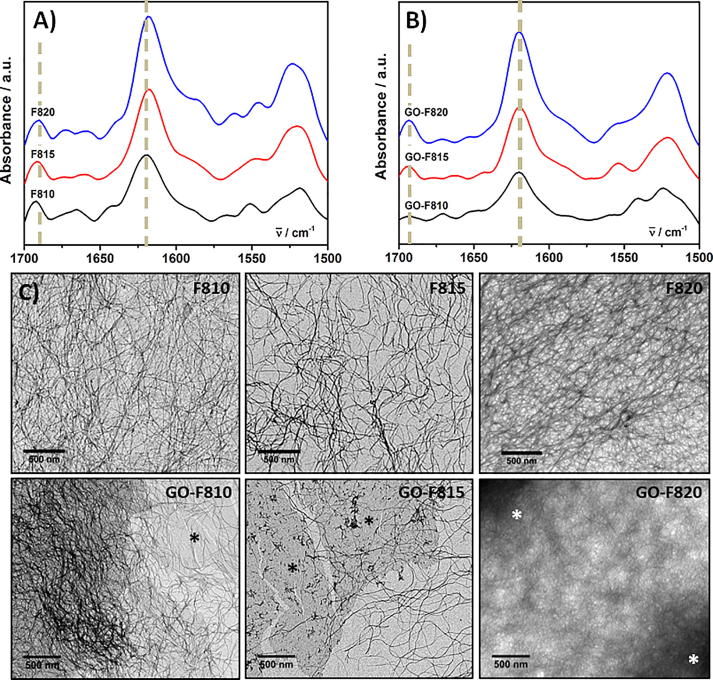
Top: FTIR spectra of peptide (A) and peptide-GO hybrid (B) hydrogels. C) TEM images of hydrogels formulated without (top row) and with (bottom row) GO. (Asterisks highlight GO flakes embedded in the peptide fibrillar matrix).

First we investigated the effect that incorporating GO in the hydrogels had on the ability of the peptide to form β-sheet fibres. In Fig. 3 the FTIR spectra of the hydrogels formulated with and without GO are presented. As can be seen all spectra are similar and in all of them a strong absorption band at ∼1620 cm^−1^ and a weaker one at ∼1690 cm^−1^ are observed. These absorption bands are characteristics of the adoption by these peptides of a cross-β-sheet conformation and the formation of extended β-sheet fibres [41], [42]. The presence of fibres was also confirmed by TEM (Fig. 3). For the 20 mg mL^−1^ samples imaging was more challenging due to the density of the fibrillar network and the difficulty when preparing the specimens, to dilute/disentangle the peptide fibres. Nevertheless in all cases long semi-flexibles fibres can be observed. For the sample containing GO the flakes where found in all cases to be fully embedded within the fibrillar network.

**Fig. 4:**
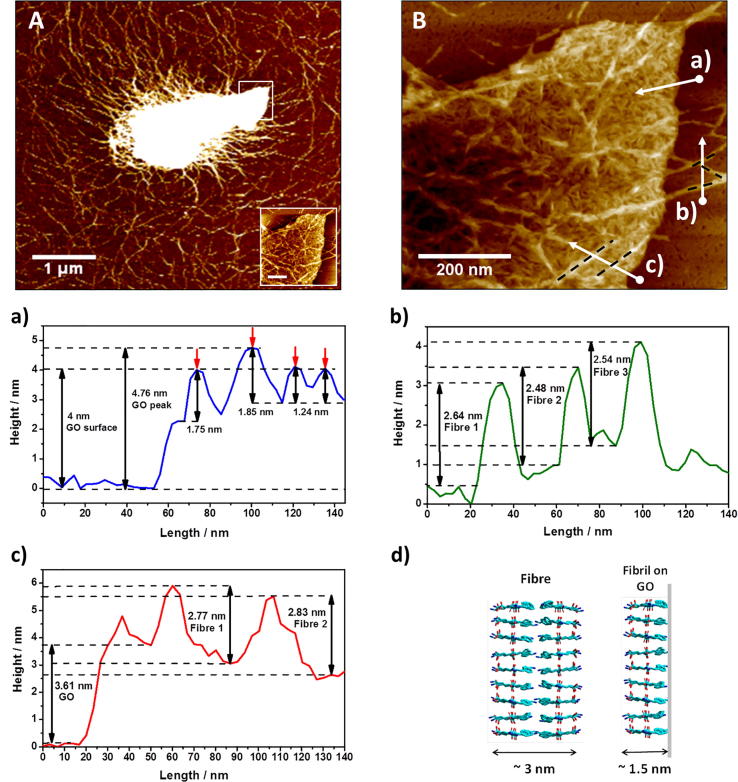
Top: AFM images of GO flakes embedded in the peptide matrix at 5 µm × 5 µm (A) and 0.7 µm × 0.7 µm (B) scan sizes. Bottom: a) to c) height profiles corresponding to the scans shown in (B) and d) schematic model of β-sheet fibres and β-sheet fibrils templated on GO surface.

In order to gain further insight into the molecular interactions between the peptide and GO high resolution AFM images were obtained of GO flakes embedded in the GO-F820 sample (Fig. 4A). As for the TEM images, the presence of a dense fibrillar network was confirmed and the GO flakes were once again found to be fully embedded within the network. A high-resolution scan of the GO flake tip was performed (Fig. 4B) and height measurements taken across the GO flake (scan a), peptide fibres (scan b) and peptide fibres deposited on top of the GO flake (scan c). For the GO flake embedded in the hybrid hydrogels the thickness was found to range between 2.5 and 4.7 nm, significantly larger than the thickness measured for isolated flakes (1.15 ± 0.04 nm Fig. ESI 2C-D). From the AFM image it is clear that the GO flake is coated (probably on both sides) by short rigid peptide fibrils with height ranging from 1.2 to 1.8 nm. These structures are reminiscent of fibrils observed for similar peptides on negatively charged mica surfaces, which we have shown to be surface templated [43]. We hypothesise therefore that these are short thin β-sheet fibrils templated on the surface of the GO flakes during hydrogel formulation. These fibrils were found to be significantly thinner than the peptide fibres themselves. Indeed from scan b the peptide fibres height was found to range from 2.5 to 2.8 nm. Peptide fibres with similar heights could also be observed deposited on top of the coated GO flakes (scan c). The difference in height between the peptide fibres and the peptide fibrils templated on the surface of the GO flake suggest different fibre structures. As discussed in our previous work, the peptide fibres are thought to be formed by two β-sheet fibrils assembling to borrow their hydrophobic surfaces [44]. The fibre heights measured are in good agreement with this model (Fig. 4d). We hypothesise that on the surface of the GO a single β-sheet fibrils forms due potentially to the phenylalanine vinyl side chains interacting with the graphitic structure of the GO flake.

These results confirm that the presence of GO does not affect the ability of this peptide to form β-sheet fibres nor hydrogels. They though highlight the presence of strong interactions between the GO flakes and the peptides that are expected to have a significant effect on the bulk mechanical properties of the hydrogels.

**Fig. 5:**
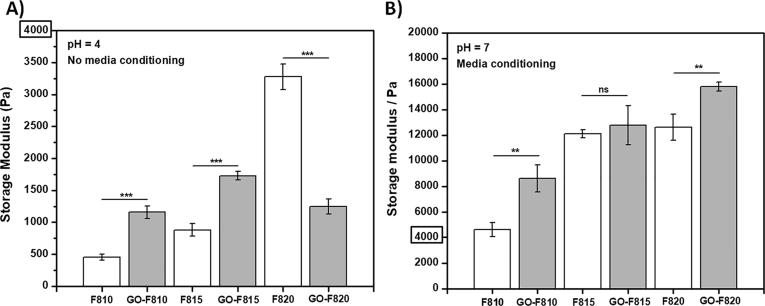
Storage moduli at 1 Hz of peptide hydrogels as formulated at pH = 4 (A) and after overnight media conditioning at pH = 7 (B). All measurements were performed at least 3 times at 37 °C. (Data are shown as mean ± SD; ^***^p-value <0.001; ^**^p-value <0.05).

Next we investigated the effect that adding GO flakes had on the shear moduli of the hydrogels after formulation (i.e.: pH = 4) and after conditioning the gel with cell culture media (i.e.: pH 7.4). The latter condition was used as these will be the mechanical properties actually experienced by the cells when encapsulated in the hydrogels. Changing the hydrogels’ pH from 4 to 7.4 will significantly alter the nature of the interactions between peptide fibres themselves as well as between peptide fibres and GO flakes, as the fibres will go from carrying a positive net-charge to becoming neutral (Fig. ESI 1). It should be noted that conditioning the hydrogels with cell culture media also introduces a variety of ions and therefore charge-screening effects will also be present [45]. Frequency sweep experiments were performed on the hydrogels in the linear viscoelastic region (LVR – Fig. ESI 4). All samples showed a solid-like behaviour across the frequency range explored (0.01–10 Hz) with the storage modulus (G′) being in each case at least one order of magnitude higher than the loss modulus (G″) (Fig. ESI 5). In Fig. 5 the shear storage moduli obtained at 1 Hz for all the samples before and after media conditioning are presented.

As expected for the peptide hydrogels prepared at pH 4 the shear modulus increases with increasing concentration as the fibrillar network density increases. When GO flakes are incorporated the shear moduli of the hydrogels were found to roughly double for the 10 and 15 mg mL^−1^ samples but to half for the 20 mg mL^−1^ sample. The detrimental effect on G′ at high peptide concentrations (>20 mg mL^−1^) of incorporating GO in these hydrogels was discussed in our previous work, where we showed that the adhesion of the peptides on the graphene derivatives was mainly driven by hydrophobic interactions [29]. The change in effect on G′ when the concentration of peptide is decreased (<20 mg mL^−1^) is tough puzzling. We hypothesise that this effect is linked to a change in the nature of the electrostatic interactions between the peptide fibrillar network and the peptide-coated GO flakes. Indeed, at pH 7 when electrostatic interactions are removed (i.e.: peptide fibres become neutral), no such effect is observed (Fig. 5B). In this system the peptide molecules can be thought as being in three different chemical states: dissolved as monomer in the water phase, self-assembled into the β-sheet fibres and deposited on the surface of the GO flakes. The amount of peptide in each state can be seen as resulting from a standard chemical equilibrium and therefore being related to the peptide chemical potential in each state. Assuming a fixed solubility for the peptide molecule in the water phase, when the peptide concentration is increased the amount of peptide self-assembled into the β-sheet fibres (i.e. fibre density) and deposited on the surface of the GO flakes (i.e.: coating density) increase. This hypothesis seems to be confirmed by the fact that at 10 and 15 mg mL^−1^ lower amount of peptides and no peptides were found to coat the GO flakes respectively (Fig. ESI 3). At low coating density the coated GO flakes will still carry an overall negative net-charge resulting in attractive electrostatic interactions with the peptide fibres. As shown in the composite literature, strong interfacial adhesion between nanofillers and matrices usually leads to improved mechanical properties [46], [47], [48], as observed here for the GO-F810 and GO-F815 samples. As the coating density increases the overall negative net-charge of the peptide-coated GO flakes will decrease, as the peptides mask the charges on the flakes’ surfaces. This process will ultimately lead to the peptide-coated GO flakes net-charge decreasing and becoming neutral or even positive. As a result a change in the nature of the electrostatic interactions between the peptide-coated GO flakes and the peptide fibres from attractive (i.e.: strong interfacial adhesion) to non-attractive/repulsive (i.e.: weak/no interfacial adhesion) occurs. The resulting weak/no interfacial adhesion (i.e.: no reinforcement) and the significant amount of peptide sequestrated on the surface of the GO flakes is thought to lead to the G′ of the GO-F820 hybrid hydrogel being lower that the G′ of the corresponding hydrogel, F820.

When conditioning with media all the hydrogels (with and without GO) were found to have significantly higher G′. The overall mechanical properties of these hydrogels is linked to the nature of the interactions between the peptide fibres which control the level of network crosslinking. At pH 4 there is a competition between hydrophobic interaction that promotes crosslinking and electrostatic repulsion that prevents crosslinking. At pH 7 the fibre do not carry a charge resulting in the elimination of the electrostatic repulsion leading to an increase in the level of hydrophobic crosslinking and a stiffening hydrogel i.e. and increase in G′. As stated above, conditioning the samples with media in addition to a change in pH also introduces significant amount of ions in the system resulting in charge screening effects being also present, once again promoting higher levels of crosslinking [45], [49]. When the GO flakes are incorporated in the hydrogels, an increase of the hydrogels G′ is observed at all peptide concentrations. At this pH the interfacial adhesion of the peptide fibres to the coated GO flakes will be mainly driven by hydrophobic interactions. As a result some level of reinforcement is observed.

**Fig. 6:**
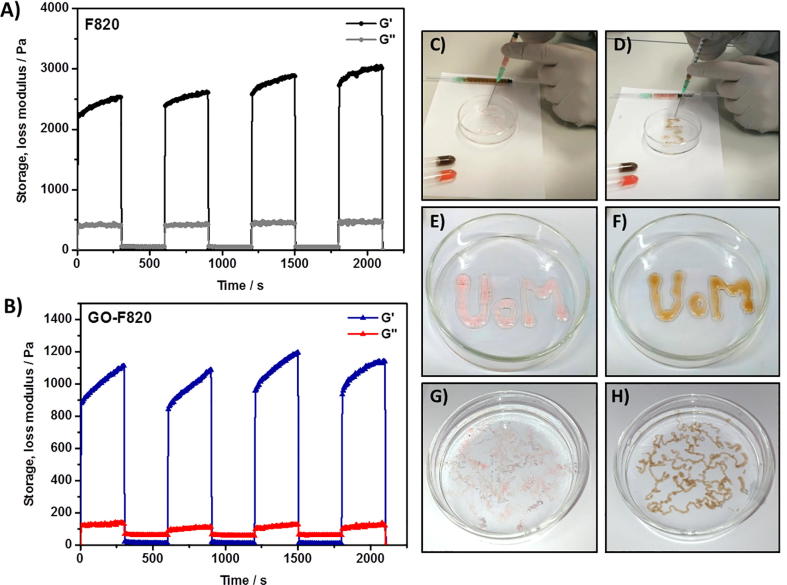
Left: Shear-thinning and recovery experiments demonstrating hydrogels’ injectability (Top: F820 formulated without GO; Bottom: GO-F820 formulated with GO). Right: Photographs of hydrogels being injected in a Petri dish (C & E: Hydrogel formulated without GO; D & F: Hydrogels formulated with GO) and in PBS (G: hydrogel formulated without GO; H: Hydrogel formulated with GO) (Pink dye was added to the hydrogels formulated without GO to enhance contrast). (For interpretation of the references to colour in this figure legend, the reader is referred to the web version of this article.)

One of the key features of these hydrogels is their injectability or in other words their shear thinning and recovery properties. In order to confirm that he incorporation of the GO did not affect the hydrogels injectability a series of experiments where performed in-situ in the rheometer during which high (hydrogel breaking) and low (hydrogel recovery) shear strains where applied successively. As can been seen from Fig. 6A-B for the 20 mg mL^−1^ samples in both cases, with and without GO, the hydrogels had the same rheological signature. When sheared at high shear strain (strain = 100%) the hydrogels shear-thin becoming liquid-like (G^″^ > G^′^) and when the shear strain is decreased (strain = 0.2%) within the LVR the hydrogels recover their solid-like behaviour (G^″^ < G^′^). This effect was found to be highly reproducible over the 3 cycles tested. In Fig. 6C–H photograph of the gels being injected in a Petri dish and in phosphate buffer saline solution at pH 7.4 (PBS was used rather than cell culture media as it is a clear solution facilitating imaging) are presented. When injected on a glass surface due to their low mechanical properties the hydrogels tend to lose shape when exiting the needle (21G) and broad hydrogels “struts” are obtained. When injecting in PBS thin “noodles”, roughly three times the size of the needle diameter, are obtained. As discussed above when the pH of the hydrogel is changed from 4 to 7.4 significant stiffening is observed. When injecting in PBS as soon as the hydrogel exits the needle the PBS diffuses in and changes its pH. As a result of this process the hydrogel stiffens and maintains a “noodle-like” shape. These results confirm that the hybrid hydrogels designed here are indeed suitable as injectable materials for cell delivery.

**Fig. 7:**
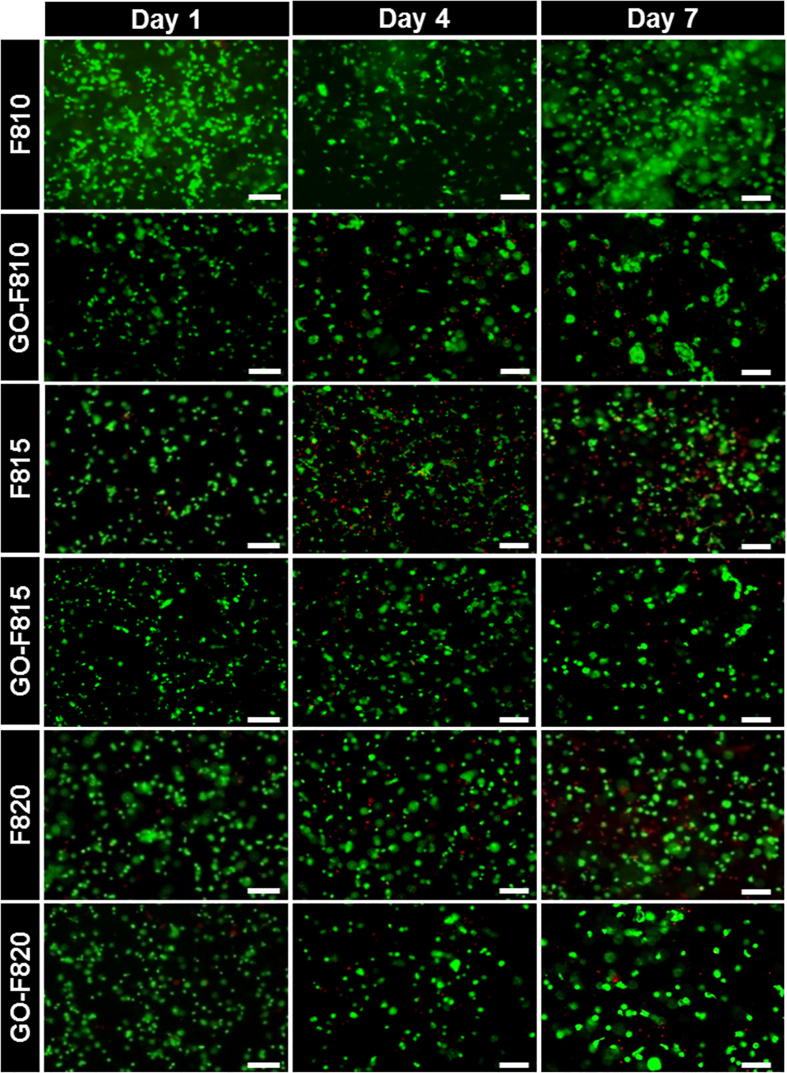
LIVE/DEAD assay (green = calcein AM for viable cells and red = ethidium homodimer-1 for dead cells) was used to assess cell viability of BNPCs. Viability was evaluated at day 1, 4 and 7 after encapsulation. Viable cells are show green fluorescence, dead cells show red fluorescence. Scale bar = 100 µm. (For interpretation of the references to colour in this figure legend, the reader is referred to the web version of this article.)

Finally, we investigated the potential of these hydrogels as delivery platform of IVD repair by culturing in 3D bovine NP cells. The cells where initially encapsulated in the hydrogel as formulated (i.e.: pH 4). The cell containing hydrogel were then plated in cell culture inserts and conditioned by surrounding the inserts with cell culture media (see Materials and Methods for further details). Cell culture was performed over a period of 7 days. In Fig. 7 live/dead assay images are presented at day 1, 4 and 7 for all the hydrogels. As can be seen mainly live cells with a characteristic round morphology could be observed in all the hydrogels, with and without GO, over the 7 days of culture.

**Fig. 8:**
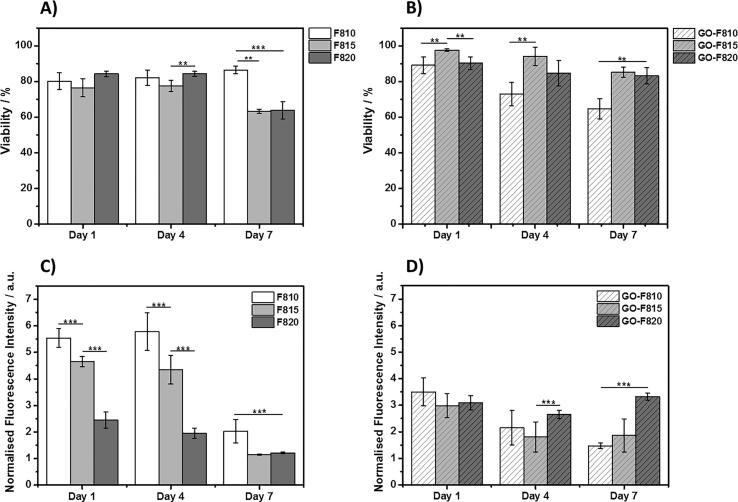
Percentage of viable cells extracted from LIVE/DEAD images for A) peptide hydrogels and B) hybrid hydrogels. Metabolic activity for cells encapsulated in C) peptide hydrogels and D) hybrid hydrogels. Data are shown as mean ± SD (^***^p-value <0.001; ^**^p-value <0.05).

In Fig. 8, cell viability and metabolic activity are presented for all the hydrogels. As can be seen, cell viability remains good in all the hydrogels. For the F810 hydrogel, viability is seen to remain high (>80%) over the 7 days of culture while for the F815 and F820 hydrogels, viability was observed to decrease from ∼80% at day 1 to ∼60% at day 7. When GO is incorporated in the hydrogels the results are somehow inverted. For the GO-F810 hydrogel, viability is observed to decrease steadily over the 7 days of culture from ∼80 to ∼60% while for GO-F815 and GO-F820 hydrogels, viability was found to remain high (>80%). As far as metabolic activities are concerned significant difference are observed in this case between hydrogel formulated with and without GO. For the hydrogel formulated without GO at day 1, the metabolic activity was found to decrease with increasing peptide concentration i.e.: with increase hydrogel stiffness. For all three samples the metabolic activity is roughly maintained at day 3 and then decreases significantly at day 7 suggesting that the cells become quiescent or senescent. When GO is incorporated in the hydrogels similar results were obtained for the GO-F810 and GO-F815 hydrogels albeit with lower starting metabolic activity levels at day 1. Interestingly for the GO-F820 sample that has a shear modulus similar to the native NP the metabolic activity was found to be slightly higher at day 1 compared to the F820 hydrogel and to remain constant over the 7 days of culture. In their native environment NP cells tend to have a low mitotic activity. High levels of cell proliferation have indeed been observed in pathological human discs subjected to mechanical stress [50] and during diseased state of IVD [51].

**Fig. 9:**
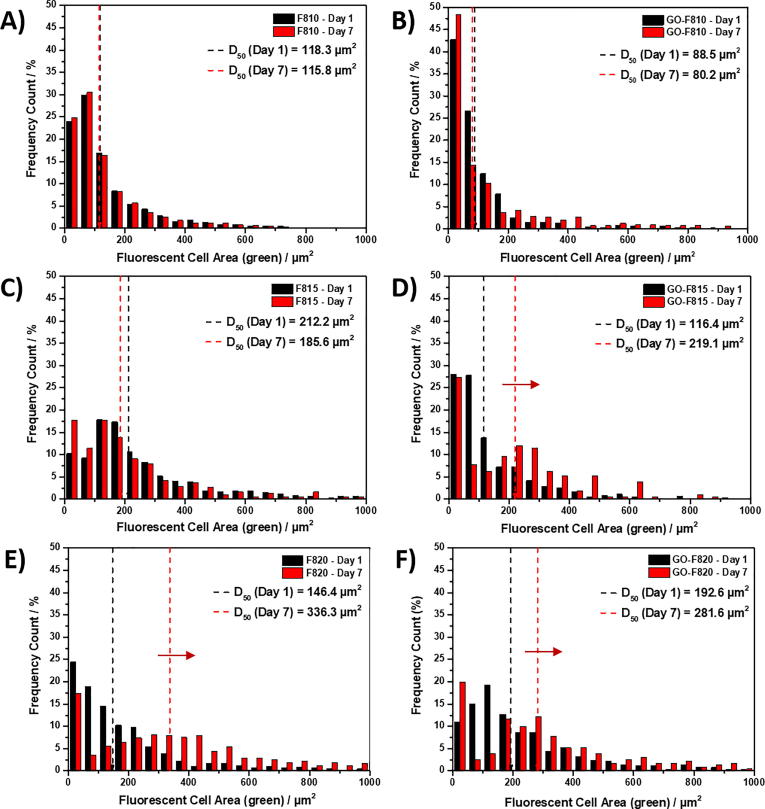
Size frequency plots of cell clusters counts obtained using LIVE/DEAD assay. A, C, E) Size frequency plots of cell clusters encapsulated in peptide hydrogels without GO, i.e. F810, F815 and F820 respectively. B, D, F) Size frequency plots of cell clusters encapsulated in peptide hydrogels with GO, i.e. GO-F810, GO-F815 and GO-F820 respectively. D50: Cell/cell cluster area value below which 50% of the cells/cell clusters area distribution is contained. Red arrows indicate shift for D50 values at day 7 compared to day 1. (For interpretation of the references to colour in this figure legend, the reader is referred to the web version of this article.)

Another characteristic of NP cells in their native environment is the tendency to cluster into small groups of cells, which have been referred as the “functional units” for disc repair, within the NP [52], [53]. In order to investigate the clustering tendency of NP cells in our hydrogels the live/dead images were used to analyse the size of the observed cell/cell clusters using an approach used to characterise the distribution of nano-particles (see materials and method for more details). The size distribution of the cell clusters and the D50 values (Cell/cell cluster area value below which 50% of the cells/cell clusters area distribution is contained) are presented in Fig. 9. For the three hydrogels, F810, F815 and GO-F810, with the lowest G′ no change in the cell cluster size distribution was observed over the 7 days of culture. On the other hand, for the stiffer hydrogels, F820, GO-F815 and GO-F820, the clusters sizes are found to increase significantly at day 7. The cluster population size is centred around 250–300 μm^2^ corresponding roughly to clusters of 15–20 cells.

The results obtained, in particular for the GO-F820 sample, clearly highlight the potential of these hybrid hydrogels as injectable scaffold for the culture and delivery of NP cells.

## 4 Conclusion

We have designed and characterised a series of FEFKFEFK-GO hybrid hydrogels. Our results clearly confirm the presence of strong interactions between this peptide and the GO flakes with the peptide coating and forming short thin fibrils on the surface of the flakes. These strong interactions affect the bulk properties of the final hybrid hydrogels, which are a function of the peptide concentration and hydrogel pH. At pH 4 electrostatic interactions between the peptide fibres and the peptide-coated GO flakes are thought to govern the final bulk properties of the hydrogels. At low peptide concentration, where attractive electrostatic interactions are present, the incorporation of GO result in an increase in the hydrogel G′ while at high peptide concentration, where electrostatic iterations are absent or repulsive, it results in a decrease in G′. At pH 7, after conditioning with cell culture media, electrostatic interactions are removed and the main interactions controlling the final properties of the hydrogels are thought to be hydrophobic. At this pH, G′ was found to increase at all concentrations upon incorporation of GO. Our results also show that the incorporation of GO does not affect their injectability.

In order to explore the potential of these new hybrid hydrogels as cell delivery platform for IVD regeneration, NP cells were culture in 3D. Our results clearly show that the incorporation of GO improve cell viability and metabolic activity, in particular the GO-F820 hybrid hydrogel promotes high cell viability and maintain cell metabolic activity over the 7 days of culture. This hydrogel has mechanical properties similar to the NP and was shown to be injectable making it a good candidate as injectable scaffold for the delivery of NP cells in the IVD.
