# Supplementary material for: Graphene oxide containing self-assembling peptide hybrid hydrogels as a potential 3D injectable cell delivery platform for intervertebral disc repair applications
Source: Acta Biomater. 2019 Jul 1;92:92–103. doi: 10.1016/j.actbio.2019.05.004 (PMC6582688; doi:10.1016/j.actbio.2019.05.004)
Supplement: Supplementary Data 1 [file mmc1.docx]

**Graphene oxide containing self-assembling peptide hybrid hydrogels as a potential 3D injectable cell delivery platform for intervertebral disc repair applications.**

Cosimo Ligorio^a,b^, Mi Zhou^a,b,±^ , Jacek K. Wychowaniec^a,b,§^, Xinyi Zhu^b,c^, Cian Bartlam^a^, Aline F. Miller^c,±^, Aravind Vijayaraghavan^a,d^, Judith A Hoyland^e,f^, Alberto Saiani^a ,b,*^

**Electronic Supplementary Information**

**Figure ESI 1**: Theoretical charge carried by the FEFKFEFK vs. pH. Vertical lines highlight pH=4 and pH=7. The theoretical charge was calculated using the following equation:

Where N_i/j_ are the numbers and pKa_i/j_ the pKa values of the basic (i - pKa > 7) and acidic
(j - pKa < 7) groups present on the peptide (pKa_lysine_ = 10.53; pKa_glutamic acid_ = 4.26; pKa_C-terminus_ = 2.18; pKa_N-terminus_ = 9.13).


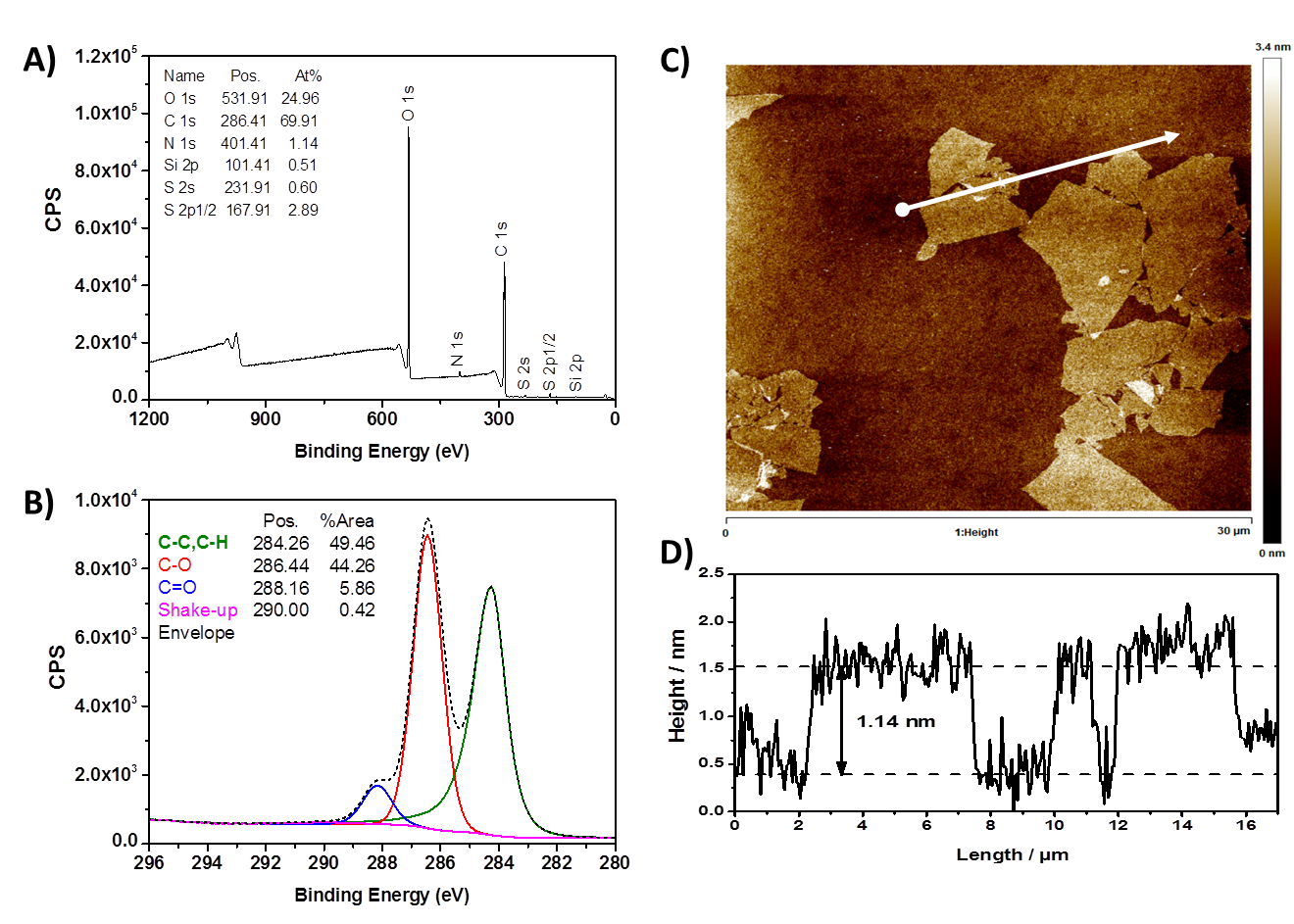


**Figure ESI 2: A)** Wide X-ray photoelectron spectroscopy (XPS) scan of GO. **B)** C1 region XPS scans of GO. XPS scans were collected using an Axis Ultra DLD spectrometer (Kratos, Manchester) equipped with a Al Kα X-ray source (1486 eV, 10 mA emission). Survey spectra and scans of atomic core levels were performed with a pass energy of 80 eV and 20 eV respectively, under vacuum (pressures < 3 × 10^−8^ mbar). XPS data were processed using CasaXPS software ([www.casaxps.com](http://www.casaxps.com)) by fitting photoelectron peaks with product-approximation using Gaussian-Lorenztian peaks. XPS spectrum was fitted with the synthetic components paying attention to minimise the total square error fit. Samples for XPS analysis were prepared by drop casting GO solution on a plasma-treated Si/SiO_2_ substrate to form a film of at least 10 nm-thick (penetration depth of XPS X-rays is approximately 10 nm). **C)** AFM micrograph of GO flakes deposited on Si / SiO_2_ substrate and **D)** height vs length distance (height profile) plot to probe GO flake thickness. See Materials and Methods for experimental details on sample preparation.


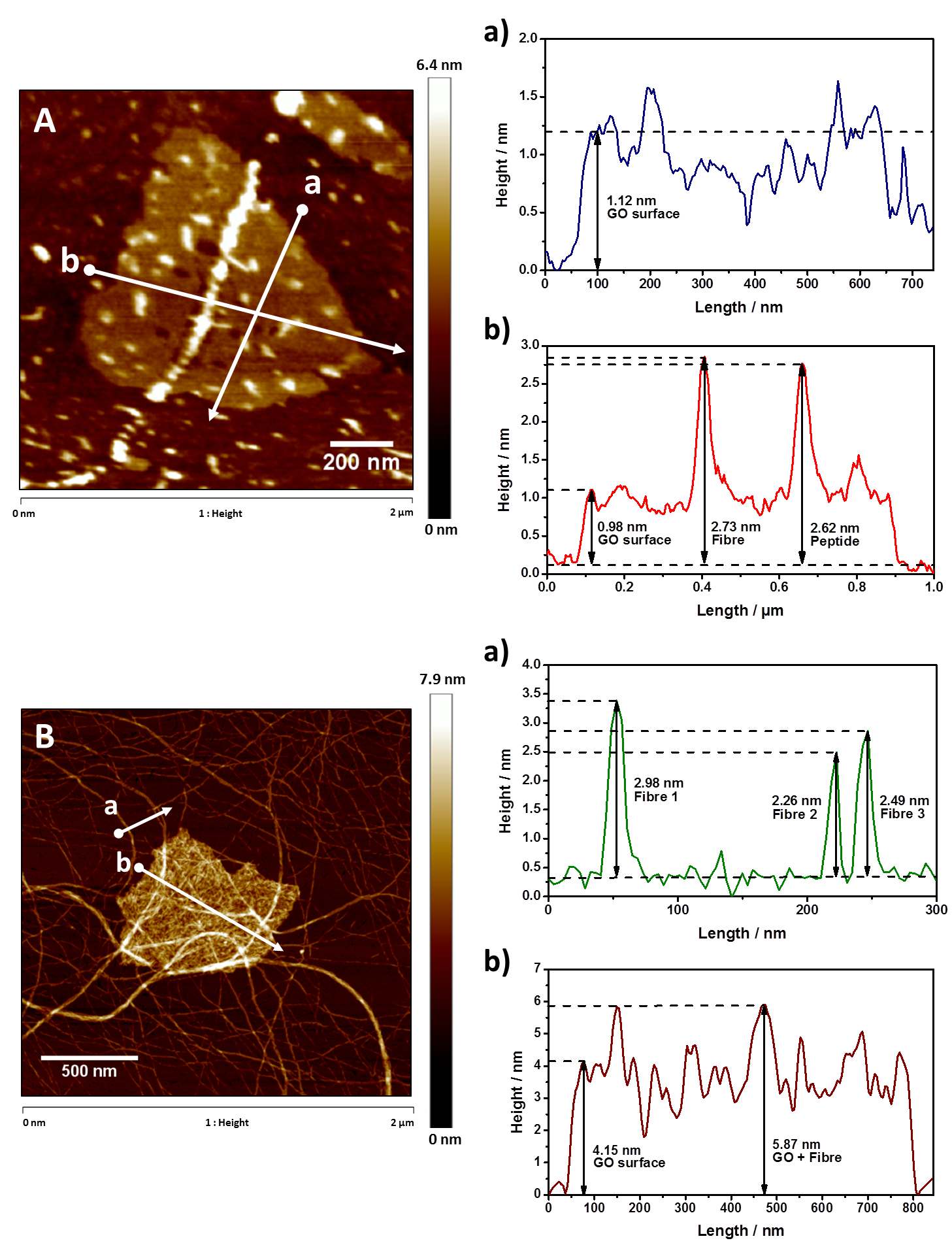


Figure ESI 3. Left: AFM micrographs of GO-F810 (A) and GO-F815 hydrogels (B). Right: height profile plots corresponding to the thickness sections indicated by white arrows.

Figure ESI 4. Oscillatory rheometry performed in amplitude sweep mode for F820 (left) and GO-F820 (right) hydrogels shows the linear viscoelastic region (LVR) of the samples (grey area).

Figure ESI 5. Oscillatory rheometry in frequency sweep mode performed on hydrogels formulated without GO (top) and with GO (bottom) before (left – pH 4), and after exposure to cell culture media (right – pH 7).
